# The Correlations Between Diabetes Mellitus and Oro-Maxillofacial Disorders: A Statistical Perspective

**DOI:** 10.3390/dj13080373

**Published:** 2025-08-18

**Authors:** Ionut Catalin Botezatu, Mihaela Salceanu, Ana Emanuela Botez, Cristina Daniela Dimitriu, Oana Elena Ciurcanu, Claudiu Topoliceanu, Elena-Carmen Cotrutz, Maria-Alexandra Martu

**Affiliations:** 1 Department of Cell and Molecular Biology, “Grigore T. Popa” University of Medicine and Pharmacy, 16 Universității Street, 700115 Iași, Romania; ionut-catalin.botezatu@umfiasi.ro (I.C.B.); ana-emanuela.botez@umfiasi.ro (A.E.B.); elena-carmen.cotrutz@umfiasi.ro (E.-C.C.); 2 Department of Odontology-Periodontology and Fixed Restorations, Faculty of Dental Medicine, University of Medicine and Pharmacy “Grigore T. Popa”, 700115 Iasi, Romania; topoliceanu.claudiu@guest.umfiasi.ro (C.T.); maria-alexandra.martu@umfiasi.ro (M.-A.M.); 3 Department of Biochemistry, “Grigore T. Popa” University of Medicine and Pharmacy, 16 Universității Street, 700115 Iasi, Romania; daniela.dimitriu@umfiasi.ro; 4 Department of Dento-Alveolar Surgery, “Grigore T. Popa” University of Medicine and Pharmacy, 16 Universității Street, 700115 Iasi, Romania; oana.ciurcanu@umfiasi.ro

**Keywords:** diabetes mellitus, periodontal disease, apical periodontitis, root remnants, malignant tumors

## Abstract

**Background/Objectives:** The goal of this research was to determine the prevalence and distribution of the oro-maxillofacial pathologies in patients with diabetes mellitus and to determine the correlation between diabetes mellitus and oro-maxillofacial disorders. **Methods**: The retrospective study group consisted of 6868 patients (mean age 49.84 ± 22.79 years) admitted in Oral and Maxillofacial Surgery Department between 2018 and 2024. Qualitative data were analyzed by Chi-square (χ^2^) test. Odds Ratio (OR) and Relative Risk (RR) were measured for each oro-maxillofacial pathology. Quantitative data were analyzed by Student’s T-test. **Results**: Among patients with diabetes mellitus (DM), the estimated risk of malignant tumors was 5.29 times higher (RR = 5.29; *p* = 0.001) compared to the non-DM group, with 15.0% of diabetic patients affected, versus 1.4% in the non-diabetic group. The probability of periodontal disease in diabetic patients was 4.66 times higher (RR = 4.66; *p* = 0.001), affecting 5.5% of the DM group, compared to only 0.6% in the non-DM group. Diabetic patients had a likelihood 3.53 times higher (RR = 3.53; *p* = 0.001) of developing apical periodontitis, with 5.3% of the DM group affected, in contrast to 1.0% of the non-DM group. The presence of root remnants was 1.43 times more likely (RR = 1.43; *p* = 0.001) in diabetic patients, with 9.0% of the DM group affected, compared to 6.1% in the non-DM group. **Conclusions**: The strong correlation between diabetes and oral pathologies, particularly malignancies and periodontal disease, underscores the need for early screening, preventive care, and targeted management strategies for diabetic patients.

## 1. Introduction

In 2007, the Executive Board of the World Health Organization (WHO) recognized the close connection between oral health, general health, and quality of life [1]. Demographic changes, including population growth and aging, have significantly increased the cumulative burden of oral diseases [2]. Epidemiological studies contribute to the development of prevention strategies both at the population level and for high-risk groups, covering a wide range of chronic diseases from the prenatal period to old age [3].

The most common conditions include dental caries, periodontal disease, and oral mucosal lesions. If left untreated, caries can progress to the dental pulp leading to infection, and tooth loss [4,5]. Periodontal disease—ranging from gingivitis to severe periodontitis—affects the supporting structures of the teeth and is driven by microbial plaque and the host’s inflammatory response. It not only leads to tooth mobility and eventual tooth loss but is also increasingly recognized for its associations with systemic conditions such as diabetes, cancer, cardiovascular, renal, autoimmune, and neurologic diseases [6,7]. Other frequently encountered oral pathologies include candidiasis, aphthous ulcers, and precancerous lesions, all of which can cause discomfort, aesthetic concerns, and functional limitations [8]. Recognizing and addressing these conditions early through preventive care and patient education is essential in promoting long-term oral and general health.

Diabetes mellitus is an increasingly significant public health issue and one of the most prevalent chronic metabolic diseases globally [9]. Diabetes mellitus disrupts barrier function and impairs healing responses, hinders keratinocyte proliferation and migration, alters inflammatory responses, and reduces the formation of new connective tissue and bone [10,11].

Diabetes-related oral complications include periodontal diseases (periodontitis and gingivitis), salivary dysfunction with reduced flow and altered composition, taste disturbances, increased susceptibility to fungal and bacterial infections, various oral mucosal lesions, as well as delayed mucosal wound healing, neurosensory disorders, dental caries, and tooth loss [11].

The aim of the research was to determine the prevalence and distribution of oro-maxillofacial pathologies in patients with diabetes mellitus and to determine the risk of the assessed pathology in diabetic patients when compared with non-diabetic patients.

## 2. Materials and Methods

The retrospective study consisted of 6868 patients who were admitted to the Oral and Maxillofacial Surgery Department (“Sfântul Spiridon” Emergency Hospital from Iași, Romania) between January 2018 and December 2024. One investigator analyzed the medical records of the patients.

Inclusion criteria:

- Oral pathology;
- Availability of complete medical and diagnostic records;
- Confirmed diagnosis regarding the presence or absence of diabetes mellitus.

Exclusion criteria:

- Incomplete or inconsistent medical records;
- Unconfirmed diabetic status;
- Patients with maxillofacial trauma or congenital craniofacial anomalies unrelated to systemic disease.

This study was conducted in strict adherence to the ethical principles outlined in the Declaration of Helsinki. The research was reviewed and approved by the Research Ethics Committee of the “Sfântul Spiridon” Emergency Hospital in Iași, under approval number 104 on 26 October 2023. Prior to participation, informed consent was obtained from all individuals included in the study.

The demographic data of the study group: gender distribution: 57.2%—males, 42.8%—females; age distribution: mean age 49.84 ± 22.79 years (range 1–97 yrs.); median age 54 yrs. (49.7% of patients > 54 yrs.; 50.3% of patients < 54 yrs.); environment of origin: 47.3%—urban; 52.7%—rural.

**Figure 1:**
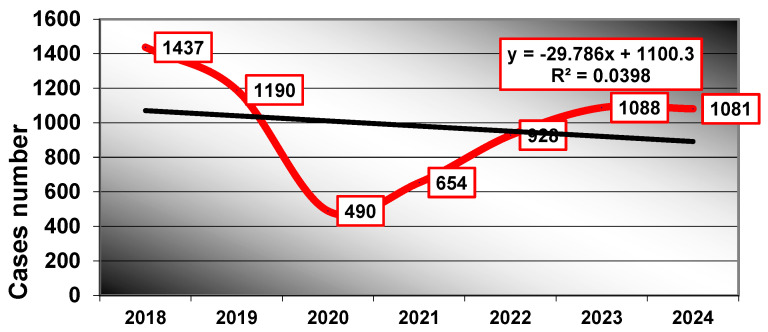
Cases frequency/follow-up years related to oral-maxillofacial pathology.

The clinical cases revealed a peak frequency in 2018 (n = 1437) and a minimum during the pandemic year 2020 (n = 490), with a decreasing trend over the period (y = 1100 − 29.79x), predicting approximately 891 cases in 2025 (Figure 1).

The assessed variables were age, gender, and environment, while the Relative Risk (RR) was calculated as a statistical measure to estimate the strength of association between diabetes mellitus and each oro-maxillofacial pathology.

Data were extracted from the hospital’s digital medical database and centralized using SPSS 18.0 software. Key variables analyzed included patient demographics (age, gender, environment), systemic diagnosis of diabetes mellitus, and presence of the following oro-maxillofacial pathologies:

- Malignant tumors (MT);
- Periodontal disease (PD);
- Apical periodontitis (PA);
- Periapical cysts (PC);
- Root remnants (R).

Diabetes mellitus was classified based on medical records confirming prior diagnosis by a specialist in internal medicine or endocrinology. The diagnosis of diabetes mellitus was confirmed based on existing medical records at the time of hospital admission, in accordance with the diagnostic criteria established by the World Health Organization (WHO): fasting plasma glucose ≥ 126 mg/dL (7.0 mmol/L), 2-hour plasma glucose ≥ 200 mg/dL (11.1 mmol/L) during an oral glucose tolerance test, or HbA1c ≥ 6.5% (48 mmol/mol), or a documented history of pharmacologically treated diabetes [12].

Oro-maxillofacial pathologies were identified based on clinical (periodontal pockets depth, bleeding of probing) and radiological diagnosis (marginal bone loss) following current guidelines of periodontal diseases (EFP/EAP classification) [13], imaging (panoramic radiographs, periapical radiographs, CBCT when applicable) (apical periodontitis, periapical cysts) [14], and clinical and histopathological reports for presence of oral malignancies [15].

**Null hypothesis** **(H_0_).** There is no statistically significant difference in the prevalence of oral and maxillofacial pathologies between diabetic and non-diabetic patients.

### Statistical Analysis

The data were compiled and analyzed using SPSS 18.0 software. Descriptive statistics included measures of central tendency (mean, median) and variability (standard deviation, confidence intervals). For the analysis of categorical variables, the Chi-square (χ^2^) test was applied to assess differences between groups. For continuous variables, Student’s *t*-test was used. The strength of associations between diabetes mellitus and specific oro-maxillofacial conditions was evaluated by calculating Odds Ratios (OR) and Relative Risks (RR). A *p*-value below 0.05 was considered statistically significant.

## 3. Results

In the study group, 925 patients (13.5%) were diagnosed with diabetes mellitus (DM group), while 5943 (86.5%) were in the non-diabetes group (NDM group).

Diabetes mellitus was diagnosed more frequently (Table 1):

- In males (58.7%; RR = 1.06; 95% CI: 0.94–1.20; *p* = 0.175);
- Slightly more frequently in individuals over 54 years of age (50.6%; RR = 1.01; 95% CI: 0.99–1.02; *p* = 0.297), with the most affected age group being 60–69 years (20.3%);
- In patients from urban areas (50.7%; RR = 1.15; 95% CI: 1.02–1.29; *p* = 0.014).

**Figure 2:**
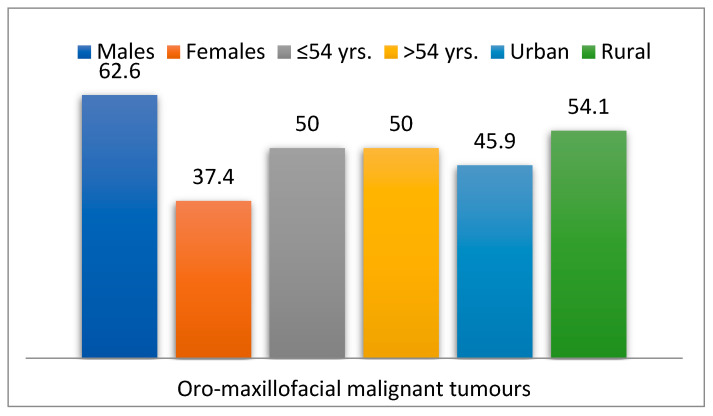
Socio-demographic features of patients with oro-maxillofacial malignant tumors.

Out of the total study group, 222 patients (3.2%) had malignant tumors in the oral and maxillofacial region (MT group), while 6646 patients (96.8%) did not have this comorbidity (non-MT group). Among the 222 patients (3.2%) with malignant tumors, 62.6% were male (*p* = 0.098), 50% were over 54 years old (*p* = 0.933), and 54.1% were from rural areas (*p* = 0.680) (Figure 2).

**Figure 3:**
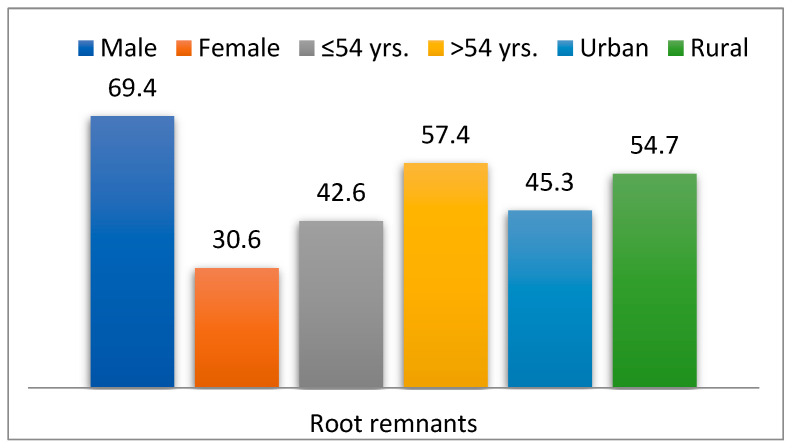
Descriptive features of patients with root remnants.

Out of the total study group, 444 patients (6.5%) had root remnants (RR group), while 6424 (93.5%) did not have this comorbidity (non-RR group). Among the 444 patients (6.5%) with root remnants, 69.4% were male (*p* = 0.001), 57.4% were over 54 years old (*p* = 0.001), and 54.7% came from rural areas (*p* = 0.201) (Figure 3).

**Figure 4:**
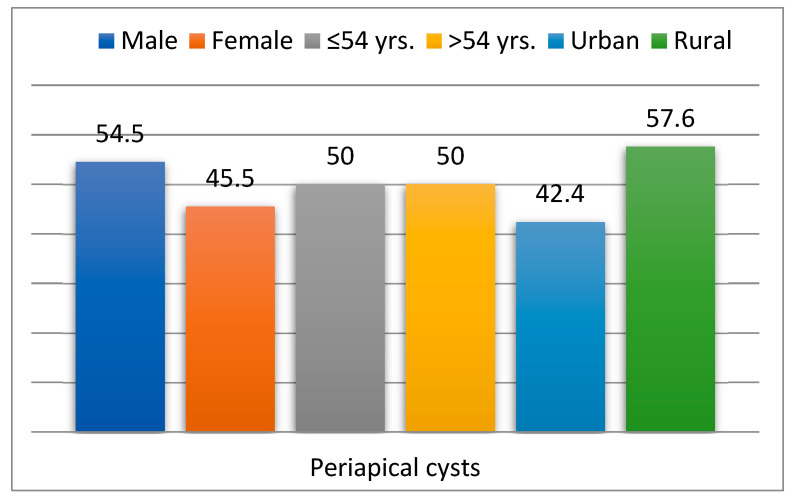
Descriptive features of patients with periapical cysts.

Out of the total study group, 66 patients (1%) had periapical cysts (CP group), while 6802 (99%) did not have this comorbidity (non-PC group). Among the 66 patients (1%) with periapical cysts, 54.5% were male (*p* = 0.373), 50% were over 54 years old (*p* = 0.531), and 57.6% came from rural areas (*p* = 0.251). In patients with diabetes mellitus, the estimated probability of having periapical cysts was 2.55 times higher (RR = 2.55; 95% CI: 1.81–3.60; *p* = 0.001) (Figure 4, Table 2).

**Figure 5:**
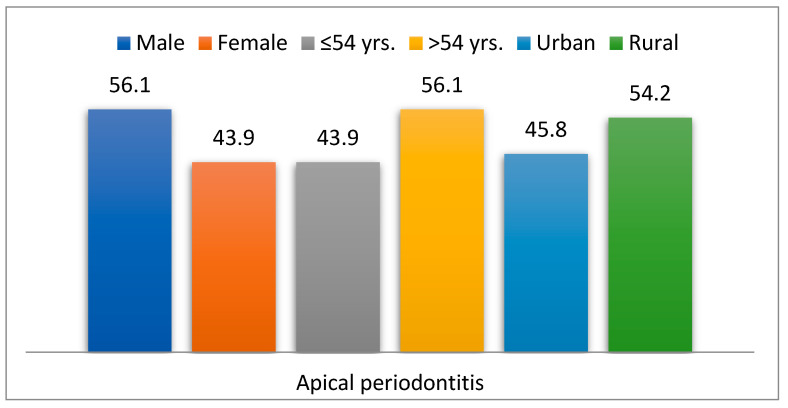
Descriptive features of patients with apical periodontitis.

Out of the total study group, 107 patients (1.6%) had apical periodontitis (PA group), while 6761 (98.4%) did not have this comorbidity (non-PA group). Among the 107 patients (1.6%) with apical periodontitis, 56.1% were male (*p* = 0.440), 56.1% were over 54 years old (*p* = 0.110), and 54.2% came from rural areas (*p* = 0.414) (95% CI: 2.85–4.38; *p* = 0.001) (Table 2, Figure 5).

**Figure 6:**
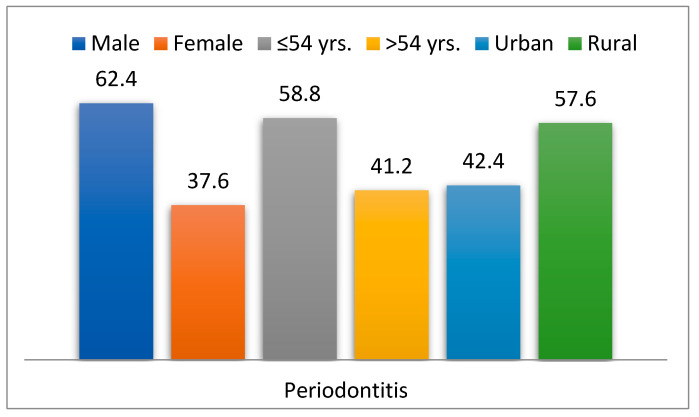
Descriptive features of patients with periodontal disease.

Out of the total study group, 85 patients (1.2%) had periodontal disease (PD group), while 6783 (98.8%) did not have this comorbidity (non-PD group). Among the 85 patients (1.2%) with periodontal disease, 62.4% were male (*p* = 0.334), 58.8% were under 55 years old (*p* = 0.070), and 57.6% came from rural areas (*p* = 0.209) (95% CI: 3.87–5.60; *p* = 0.001) (Table 2, Figure 6).

Table 2 exposes the risk of pathology in diabetic patients when compared with the control group (non-diabetic patients). In patients with diabetes mellitus, the estimated risk of malignant tumors was 5.29 times higher (RR = 5.29; 95% CI: 4.69–5.98; *p* = 0.001) compared to the non-DM group, with 15.0% of diabetic patients affected, versus 1.4% in the non-diabetic group. The probability of periodontal disease in diabetic patients was 4.66 times higher (RR = 4.66; 95% CI: 3.87–5.60; *p* = 0.001), affecting 5.5% of the DM group, compared to only 0.6% in the non-DM group. Diabetic patients had a likelihood 3.53 times higher (RR = 3.53; 95% CI: 2.85–4.38; *p* = 0.001) of developing apical periodontitis, with 5.3% of the DM group affected, in contrast to 1.0% of the non-DM group. The risk of periapical cysts in diabetic patients was 2.55 times higher (RR = 2.55; 95% CI: 1.81–3.60; *p* = 0.001), occurring in 2.4% of the DM group compared to 0.7% in the non-DM group. Lastly, the presence of root remnants was 1.43 times more likely (RR = 1.43; 95% CI: 1.16–1.75; *p* = 0.001) in diabetic patients, with 9.0% of the DM group affected, compared to 6.1% in the non-DM group.

## 4. Discussion

Diabetes is a major health issue worldwide with significant consequences on the overall systemic health [6]. The oral effects of this pathology are discussed in the preset study. Diabetes is an important risk factor for periodontal disease, as hyperglycemia promotes chronic inflammation, affecting the supporting structures of the teeth and accelerating the progression of periodontitis. This relationship is two-way—while high blood sugar can worsen periodontitis, the presence of this oral disease can also make it harder for diabetic patients to keep their blood glucose levels under control [16,17]. In our research, the study group consisted of 6868 patients, of which 13.5% had diabetes mellitus, more frequently observed in males, aged over 54 years, from urban areas. In the study group of diabetic patients, the risk of developing various oral and systemic conditions was significantly higher compared to non-diabetic individuals. This result sustains the literature data [11].

In our study the probability of periodontal disease was 4.66 times higher, occurring in 5.5% of the diabetic group versus 0.6% in the non-diabetic group. Other research groups reported 3 times higher risks of periodontal disease in diabetic patients [18,19]. Epidemiological studies indicate an increased incidence of periodontitis in individuals with uncontrolled diabetes, suggesting that this condition may be considered a complication of diabetes. The association between diabetes mellitus and periodontal disease is driven by multiple mechanisms, including the release of advanced glycation end-products due to hyperglycemia, along with a range of shared predisposing factors, such as genetic susceptibility, microbial influences, and lifestyle habits. Periodontal infections further impair glycemic control, which in turn worsens periodontal disease [20,21,22].

A dose–response relationship has been established between the severity of periodontitis and the presence of diabetes complications. Maintaining oral health is essential for diabetes management and the prevention of systemic complications, while additionally, periodontal treatment can help improve glycemic control [23,24,25,26]. Preshaw et al. (2011), in their review of the literature on the relationship between diabetes and periodontal disease, highlight patients with poorly controlled diabetes and a higher risk of developing periodontitis [19]. As a result, it is crucial to raise awareness among diabetic patients about this increased susceptibility. Early referral of individuals with poorly controlled diabetes to dental specialists for periodontal screening is strongly recommended.

Research indicates that periodontal treatment in diabetic patients can enhance glycemic control, contributing to an approximate 0.4% reduction in HbA1c levels, which may have meaningful clinical benefits in diabetes management. Regular dental check-ups should be integrated into the standard care routine for diabetic patients [20]. A comprehensive approach should incorporate lifestyle modifications, patient education, oral health education and appropriate treatment, and self-management strategies, to improve overall outcomes [27,28,29,30,31].

While the prevalence of oral cancer in patients with diabetes was 0.25% (250 per 100,000 patients with diabetes mellitus), the chance of oral cancer among patients with diabetes mellitus was 1.4 times higher compared with non-diabetic patients as reported in the literature [32]. One of the most important findings of this study is the significantly higher risk of malignant tumors in diabetic patients—more than five times greater compared to those without diabetes. Given the seriousness of such conditions, this association deserves close attention. Diabetes creates an unfavorable biological environment, marked by chronic inflammation, oxidative stress, and weakened immune responses, which can contribute to the development and progression of oral cancers. Moreover, slower healing and reduced defense mechanisms may worsen the course of the disease once it appears [32]. These findings highlight the importance of including regular screening for malignant lesions as part of routine care for diabetic patients, through close collaboration between dental professionals and other healthcare providers involved in managing chronic diseases.

Endodontic lesions are more prevalent in diabetic patients, and their treatment is more complex and costly [33,34,35,36,37,38]. The prevalence of apical periodontitis was higher in the diabetic group compared to the non-diabetic group (13.5% vs. 11.9%, respectively). Additionally, diabetic patients had a significantly greater proportion of teeth that had undergone endodontic treatment compared to non-diabetic individuals (4.18% vs. 1.82%, *p* = 0.001) Among diabetic patients, those with poorly controlled diabetes had a significantly higher prevalence of AP lesions compared to those with well-controlled diabetes [36,37,38].

The presence of root remnants was 1.43 times more likely in diabetic patients, affecting 9.0% of the DM group compared to 6.1% in the non-DM group. In adult patients, systemic diseases can affect the onset and progression of dental caries through various mechanisms. These include alterations in saliva composition, changes in enamel structure, shifts in the oral microbial flora, and modifications in the body’s immune response [39,40]. The updated classification system for periodontal disease incorporates a comprehensive risk assessment, allowing for the inclusion of new risk factors and providing a flexible framework for future revisions [41,42]. In this context, while some risk factors can be modified, others remain non-modifiable, posing a challenge in disease management, as risk is not solely dependent on the individual characteristics of the patient [43].

Additionally, our results provide further evidence to the emerging acknowledgment that the systemic complications of diabetes mellitus, resulting from its multiple pathophysiological mechanisms, go beyond classical metabolic disorders. Variability of glucose and other risk factors (blood pressure, plasma lipids, heart rate, body weight, serum uric acid) may also contribute to the development of complications of diabetes [44,45,46,47]. Oxidative stress is crucial in the pathogenesis of types 1 and 2 diabetes mellitus and their complications, and it is a common ethiopathogenic link to various oral diseases, including periodontitis [48]. However, care quality and target achievement impact risk factor variabilities in patients with diabetes mellitus [49,50,51,52]. Moreover, chronic low-grade inflammation is seen in diabetes, along with immune dysregulation and vascular changes creating a systemic environment that predisposes individuals to both oral infections and delayed healing [53,54,55,56]. Ultimately, the high prevalence of apical periodontitis and periapical cysts in our diabetic subgroup might be explained by this.

Oral diseases, in particular periodontitis and periapical infections, must be regarded as components of the systemic inflammatory burden of diabetes rather than as localized phenomena [57,58,59]. However, the interrelationship between periodontal disease and diabetes is mutual in that poor glycemic control influences periodontal disease and vice versa; thus, these chronic conditions must be managed in an interdisciplinary manner to ensure, where possible, that dental practitioners and physicians work together to maintain control over periodontal and glycemic parameters [60,61]. Furthermore, certain adjuvant therapies such as photoactivation, laser, platelet derivatives, and natural compounds can prove beneficial in aiding the favorable evolution of both diabetes and oral pathologies [62,63,64].

Behavioral factors influence both oral health maintenance and diabetes self-management, presenting opportunities to enhance the management of both conditions. Since many patients visit their dentist regularly, dentists and dental hygienists are often in a key position to detect diabetes early [65,66,67,68,69,70]. Dental professionals possess significant expertise in guiding patients toward behavioral modifications, making them a valuable yet underutilized resource in supporting healthcare teams involved in diabetes care [69,70,71].

One of the main strengths of this study is the large number of patients included—nearly 7000—which offers a solid foundation for drawing reliable conclusions. The study captures real-world clinical data from a major oral and maxillofacial surgery center, reflecting the actual health status and needs of patients. This makes the results highly relevant for daily medical and dental practice. The fact that the data allowed for a direct comparison between diabetic and non-diabetic individuals added depth to the analysis and helped highlight important differences in risk. The statistical tools used (such as Relative Risk and Odds Ratio) provided meaningful insight into how much more likely diabetic patients are to develop certain conditions. Ultimately, this study adds valuable evidence to the growing recognition that diabetes has a significant impact not only on general health, but also on oral health, underlining the need for closer collaboration between medical and dental professionals.

Limitations of the study also reflect some of the practical challenges we encountered during data collection. One of the main limitations was the lack of complete medical records for many patients. Important details such as past or current medications, smoking habits, body mass index (BMI), or the presence of other chronic diseases were often missing or inconsistently recorded. Since these factors can significantly influence both general and oral health, their absence makes it more difficult to draw firm conclusions or to identify potential risk factors with precision. Another important limitation was the absence of laboratory analyses. We did not have access to blood tests or other systemic markers of inflammation, which could have helped us better understand the underlying health status of the patients. Similarly, we were unable to collect and analyze local samples—such as saliva or plaque—that might have revealed valuable information about oral bacteria or specific biomarkers related to inflammation or infection. These highlight the importance of more comprehensive data in future studies. Incorporating both medical history and biological testing would offer a more complete picture and help us better understand the complex interactions between oral and general health.

Future research should aim to explore the mechanisms behind the observed associations in greater depth, ideally through prospective, longitudinal studies that monitor patients over time. Including clinical variables such as glycemic control, duration of diabetes, medication use, and lifestyle habits like smoking or diet would offer a more nuanced understanding of risk. It would also be valuable to incorporate laboratory analyses—such as systemic inflammatory markers, salivary biomarkers, and microbial profiles—to better understand the biological pathways involved.

## 5. Conclusions

Malignant tumors had the highest estimated risk in diabetic patients, despite their lower overall prevalence. Root remnants were the most common pathology, while periodontal disease was associated with a significant increase in risk, highlighting its strong link with diabetes. Apical periodontitis and periapical cysts also showed notably elevated risks, reinforcing the impact of diabetes on inflammatory and infectious dental conditions. The strong correlation between diabetes and oral pathologies, particularly malignancies and periodontal disease, underscores the need for early screening, preventive care, and targeted management strategies for diabetic patients. Regular dental check-ups and treatments should be emphasized for diabetics to mitigate risks of severe oral and maxillofacial complications.

## Figures and Tables

**Table 1:** Demographic features of DM and NON-DM groups.

Demographic Features	Diabetes Mellitus Group (n = 925)		Non-Diabetes Group (n = 5943)		Chi2 Test *p*	RR	95% IC
	n	%	n	%			
Gender					0.175		
Males	543	58.7	3388	57.0		1.06	0.94–1.20
Females	382	41.3	2555	43.0		0.99	0.97–1.01
Age groups					0.297		
≤54 years.	457	49.4	2996	50.4		0.97	0.86–1.09
>54 years.	468	50.6	2947	49.6		1.01	0.99–1.02
Environment of origin					0.014		
Urban	469	50.7	2780	46.8		1.15	1.02–1.29
Rural	456	49.3	3163	53.2		0.98	0.96–1.00

**Table 2:** Risk of pathology associated with diabetes mellitus.

Comorbidities	DM Group (n = 925)		Non-DM Group (n = 5943)		Chi2 Test *p*	RR	95% CI
	n	%	n	%			
Malignant tumors	139	15.0	83	1.4	0.001	5.29	4.69–5.98
Root remnants	83	9.0	361	6.1	0.001	1.43	1.16–1.75
Periapical cysts	22	2.4	43	0.7	0.001	2.55	1.81–3.60
Apical periodontitis	49	5.3	58	1.0	0.001	3.53	2.85–4.38
Periodontal disease	51	5.5	34	0.6	0.001	4.66	3.87–5.60

## Data Availability

The raw data supporting the conclusions of this article will be made available by the authors on request.
